# Effects of exogenous lipids on gelling properties of silver carp surimi gel subjected to microwave heating

**DOI:** 10.1002/fsn3.3021

**Published:** 2022-08-09

**Authors:** Yiling Yang, Linglu Meng, Yuxin Wang, Bowen Yan

**Affiliations:** ^1^ State Key Laboratory of Food Science and Technology Jiangnan University Wuxi China; ^2^ School of Food Science and Technology Jiangnan University Wuxi China

**Keywords:** exogenous lipid, gel property, microwave, surimi

## Abstract

Lipids are important components of surimi products because they enhance the whiteness and flavor of food. The effects of three common types of exogenous lipids on the gel properties of surimi subjected to two different heating methods were investigated in this work, using frozen silver carp (*Hypophthalmichthys molitrix*) surimi as the raw material. The surimi gels were prepared by two‐stage water bath heating or single‐stage water bath heating followed by microwave heating. We found that the quality of surimi gels was in the order of lard > chicken fat/soybean oil, which may be associated with polyunsaturated fatty acid content. The surimi gel strength was reduced with an increase in the amount of lipid added. Microwave heating significantly increased the gel strength of surimi containing exogenous lipids when compared to conventional heating. Surimi gels prepared by microwave heating showed more denser protein network microstructures by scanning electron microscopy (SEM), suggesting aggregation of protein molecules. The findings of this study provide a theoretical basis for using microwave heating to generate surimi gels with exogenous lipids.

## Graphical abstract

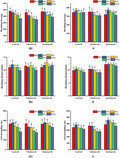

## 1 INTRODUCTION

Frozen surimi products are one of the frozen food products that are very common in our life (Luo et al., 2008). The processing of surimi typically involves multiple washing treatments for the removal of lipid components and blood from fish meat, as well as to reduce the odor (Poowakanjana & Park, 2013). Washing also enables the condensation of proteins and effectively prevents the possible oxidation of surimi products during storage, which may otherwise cause the deterioration of surimi quality (Sampels, 2015). During subsequent surimi processing, exogenous lipids (such as lard, chicken fat, and vegetable oil) are often added to maintain the unique texture and flavor of surimi (Jiao et al., 2019). Studies have demonstrated that the fat content of surimi affects its subsequent storage quality, overall textural properties, sensory quality, water retention ability, and decreases the gel strength of surimi products (Hsu & Chiang, 2010; Jiao et al., 2019; Pietrowski et al., 2011; Shi et al., 2014; Zhou et al., 2017). Other studies have shown that microwave heating is very efficient, which allows rapid traversal of the gel deterioration temperature range and prevents protein degradation, thereby enabling the appropriate enhancement of surimi gel strength (Cao et al., 2018a,b,^2020^; Jiao et al., 2021; Meng et al., 2021; Yan et al., 2020). However, existing research has primarily focused on the effects of a single type of vegetable oil on surimi gel quality, with few studies examining the effects of different types and amounts of added exogenous lipids on surimi gel quality (Gani & Benjakul, 2018; Gani et al., 2018; Okazaki et al., 2006; Pérez‐Mateos et al., 2004).

Most researchers have used conventional two‐stage water bath heating to study the effects of exogenous lipids on surimi gel quality, and there have been few reports on the use of microwave heating, despite its particular advantages in surimi quality assurance (Jiao et al., 2019). Therefore, two different surimi cooking methods (two‐stage water bath heating versus single‐stage water bath heating followed by microwave heating) were employed to investigate the effects of three common types of exogenous lipids on the properties of surimi gels and determine the roles served by different amounts of added exogenous lipid types in the gelation process. Our findings offer a theoretical basis for the commercialization of surimi products.

## 2 MATERIALS AND METHODS

### 2.1 Materials

Frozen surimi (stored and transported at–18°C or lower, AA grade) of freshwater fish derived from silver carp (*Hypophthalmichthys molitrix*) was purchased from Hubei Hongye Aquatic Products Co., Ltd.; plastic sausage casings were purchased from Shuanghui Foods Co., Ltd.; refined flaky salt was purchased from Jiangsu Salt Industry Group Co., Ltd.; lard and chicken fat were purchased from Shuanghui Foods Co., Ltd.; soybean oil was purchased from Shanghai Fulinmen Foods Co., Ltd.; Miaojie brand cling film was purchased from Top Household Goods Co., Ltd.

### 2.2 Preparation of surimi paste

Frozen surimi was used as the raw material for the preparation of surimi paste. Prior to kneading, the frozen surimi was removed from storage in a freezer at–18°C and thawed at 4°C for 10 h. The water content of the surimi was measured by ambient pressure drying in an oven at 105°C. The thawed surimi was cut into small pieces and stored in self‐sealing bags (600 g per bag) in a refrigerator at 4°C before use. After the installation of a bowl chopper, iced water was supplied to the outer chamber to maintain a low‐temperature environment during the chopping process. The surimi was then placed in the bowl chopper and chopped for 2 min. After the addition of 3% table salt and a certain volume of deionized water to bring the water content to 78%, the surimi was chopped for another 3 min. Lard, chicken fat, and soybean oil at 0, 3, 6, 9, and 12 g/100 g surimi were separately added into the surimi paste; composite oils obtained by mixing lard, chicken fat, and soybean oil in the ratios of 5:2:2, 2:5:2, 2:2:5, and 3:3:3 were also separately added to surimi samples. Each surimi sample was chopped for an additional 2 min before being transferred to a self‐sealing bag, stuffed into a sausage casing using a sausage stuffer, and sealed with a sealing machine. The prepared surimi sausages were stored in a refrigerator at 4°C before further use.

### 2.3 Preparation of surimi gels

Surimi gels were prepared using two different heating methods (Cao et al., 2018a). Surimi sausages were heated at 40°C for 30 min during the first stage of the typical two‐stage water bath heating method before being subjected to the second stage of heating at 90°C for 20 min immediately following the completion of the first stage of heating. After being thoroughly cooked, the surimi sausages were immediately immersed in iced water to cool down.

The second heating method involved the use of microwave heating to replace the second stage of water bath heating. During the first stage, heating was performed at 40°C for 30 min. After the completion of the water bath heating, the sausage casing was immediately removed, and the surimi content was chopped into long segments weighing 120 g each. The long segments were microwave‐heated at a power of 5 W/g for 96 s, with heating performed intermittently at intervals of 24 s each. At the end of the heating process, the surimi sausages were promptly immersed in iced water for cooling.

### 2.4 Texture profile analysis (TPA) of surimi gels

Surimi sausages were removed from storage in a refrigerator at 4°C and subjected to texture profile analysis (TPA) using the method described by (Yin & Park, 2014) with slight modifications. The surimi sausages were cut into 25‐mm‐long segments (sausage casing removal was required for surimi sausages cooked by two‐stage water bath heating). Textural parameters, such as hardness, springiness, chewiness, resilience, and adhesiveness, were determined using a TA‐XTPlus texture analyzer. The following were the operating conditions of the analyzer: probe model P/36R, pretest speed of 1 mm/s, test speed of 1 mm/s, posttest speed of 2 mm/s, compression ratio of 20%, interval time of 5 s, and trigger force of 10 g. Each set of samples was subjected to three parallel tests.

### 2.5 Measurement of gel strength of surimi gels

Gel strength was measured using the method described by (Zhang et al., 2015) with slight modifications. Surimi sausages were cut into 25‐mm‐long segments. As gel strength cannot be measured directly, a texture analyzer was used to measure breaking force and breaking distance. The operating conditions of the analyzer were as follows: 5 mm spherical probe (P/5 s), pretest speed of 1 mm/s, test speed of 1 mm/s, posttest speed of 2 mm/s, compression distance of 15 mm, and trigger force of 10 g. The force and distance corresponding to the first peak were the breaking force and breaking distance, respectively, and gel strength was calculated as follows:

(1) $$\mathrm{Gel}\;\text{strength}\;\left(g\times \mathrm{cm}\right)=\text{breaking force}\;\left(g\right)\times \text{breaking distance}\;\left(\mathrm{cm}\right)$$

Three parallel tests were performed for each set of samples, and the result for each parameter was determined by taking the average of the three test values.

### 2.6 Measurement of water holding capacity (WHC)

Water holding capacity (WHC) was measured in accordance with the method described by (Meng et al., 2021). Surimi gels were sliced into 2‐mm‐thick slices (approximately 5 g each), spread out on filter paper, and weighed precisely (M1). The gel slices were then wrapped with four layers of filter paper, placed in a centrifuge tube, and centrifuged at 4°C and 5000 r/min for 20 min. Immediately after centrifugation, the gel slices were removed from the centrifuge tube and the residual water on the gel surfaces was dried with the filter paper. The dried gel slices were accurately weighed to obtain the postcentrifugation gel mass (M2). The WHC was calculated using the following formula:

(2) $$\mathrm{WHC}\left(\%\right)=M2/M1\times 100\%$$

### 2.7 Measurement of surimi gel whiteness

Surimi sausage samples were sliced into 10‐mm‐thick slices and the L*, a*, and b* values of the samples were measured using a CR‐400 chroma meter, as described by (Benjakul et al., 2005). Three parallel measurements were made for each set of samples, and whiteness was calculated using the following formula:

(3) $$\text{Whiteness}=100-\left[\left(100-L^{*}\right)2+\left(a^{*}\right)2+\left(b^{*}\right)2\right]½$$

where L* = brightness, a* = degree of red or green component (positive value: red; negative value: green), and b* = degree of yellow or blue component (positive value: yellow; negative value: blue).

### 2.8 Observation of microscopic structures of surimi gels

Surimi gel samples were sliced into 3 mm × 2 mm slices, fixed in a 3% glutaraldehyde solution at 4°C for 24 h, washed multiple times in 0.1 mol/L phosphate‐buffered saline (pH 7.2), dehydrated through a graded series of ethanol solutions (50%, 70%, 90%, 100%), critical point freeze‐dried, gold‐sputtered using an ion beam sputter coater, and finally, observed and photographed under a scanning electron microscope (Cao et al., 2018c).

### 2.9 Fatty acid analysis of lard, chicken fat, and soybean oil

The fatty acid analysis of the exogenous lipids used in this study was performed using gas chromatography–mass spectrometry (GC–MS) on a Rtx‐WAX capillary column in the constant flow mode with the following operating parameters: carrier gas (helium) flow rate of 1.0 ml/min, split ratio of 10, and injection port temperature of 240°C (Wei et al., 2012). The following 51‐min temperature ramp program was employed for temperature ramping during the experiment: hold at 40°C for 5 min, ramp to 120°C at a rate of 20°C/min, ramp to 190°C at a rate of 5°C/min, hold for 5 min, ramp to 220°C at a rate of 5°C/min, hold for 17 min (duration of entire heating process: 51 min). MS data were collected at a scan range of 50–550 m/z, with the ion source and interface temperatures being 220 and 250°C, respectively.

### 2.10 Statistical analysis

Microsoft Office Excel 2013 and Origin 8.5 were used to compute and statistically analyze experimental data. All data were expressed as mean ± standard deviation. GC–MS was used to examine the fatty acid content. Duncan's multiple range test was used to evaluate significant differences between the groups (*p* < .05).

## 3 RESULTS AND DISCUSSION

### 3.1 Effects of exogenous lipid addition on surimi gel strength

**FIGURE 1:**
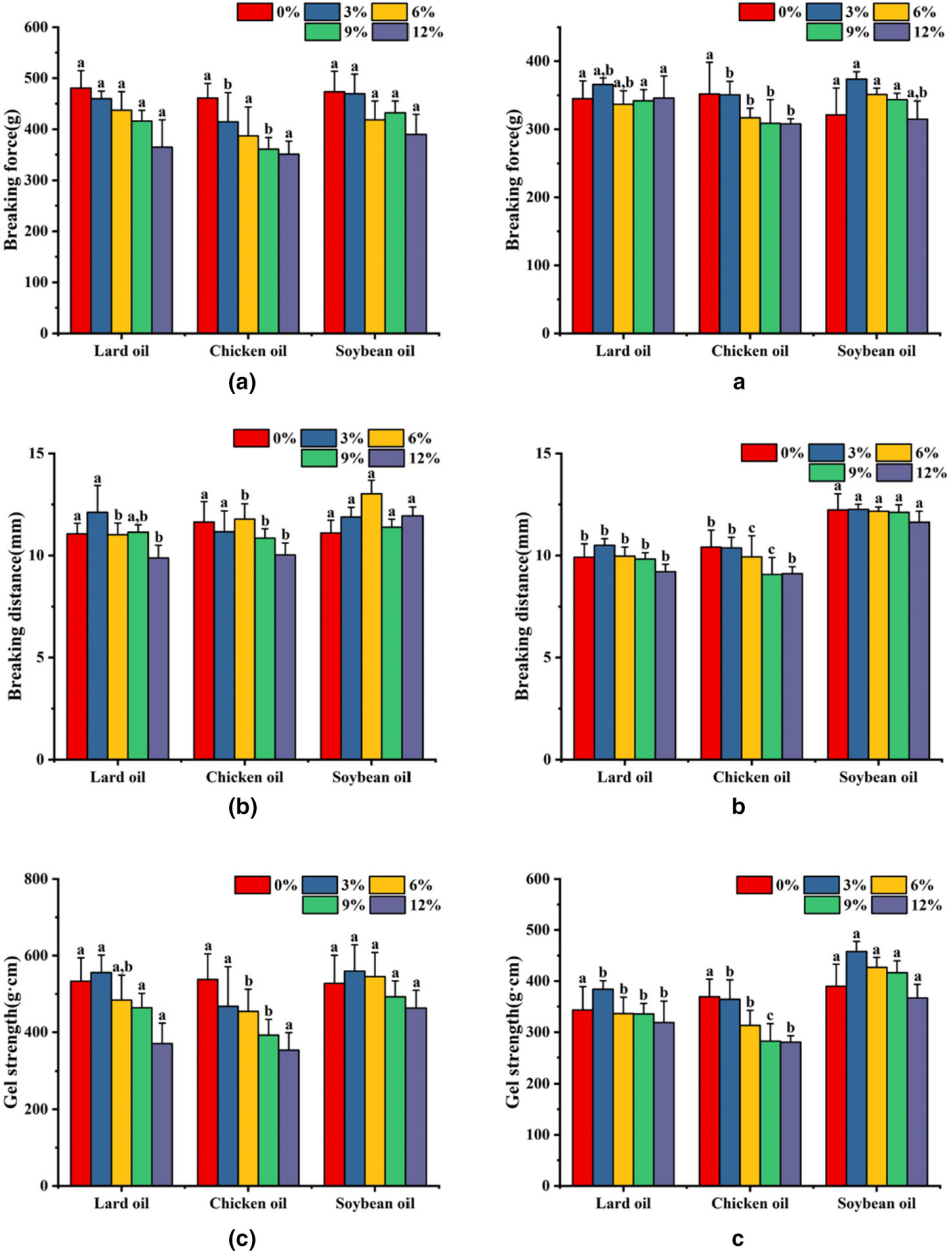
The effect of different exogenous lipids on the breaking force (A,a), breaking distance (B,b), and gel strength (C,c) of surimi gels prepared using the two different heating procedures. (A,B,C) Microwave heating, (a,b,c) water bath heating. Different letters within each color bar denote significant difference (*p* < .05) for 0%, 3%, 6%, 9%, and 12% addition of different exogenous lipids, respectively

**FIGURE 2:**
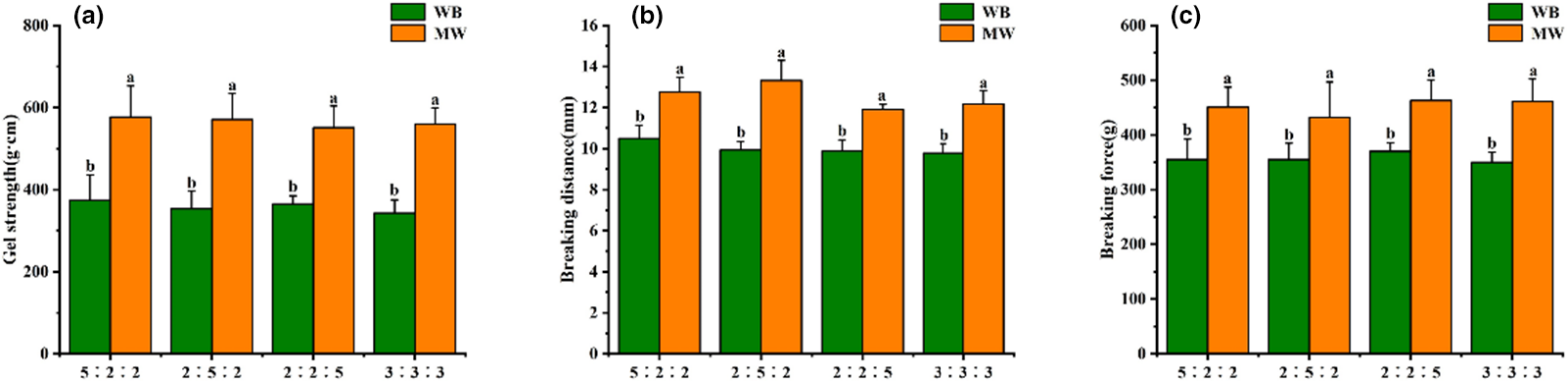
The effect of composite oils on the breaking force (a), breaking distance (b), and gel strength (c) of surimi gels prepared using the two different heating procedures. Different letters within each color bar denote significant difference (*p* < .05) between different heating treatments

Different exogenous lipids have different effects on the breaking force of surimi gels prepared using the two different heating procedures, as illustrated in Figure 1. The addition of soybean oil led to a modest increase in breaking force when compared to the addition of lard or chicken fat, whereas the breaking force values of surimi gels with added lard and chicken fat did not differ significantly. Breaking force decreased significantly as the amount of exogenous lipid increased in surimi gels prepared by microwave heating. Figure 1 also reveals that the addition of lard and chicken fat had a smaller influence on breaking distance, whereas the addition of soybean oil had a slightly higher impact. With an increase in the amount of added exogenous lipid, we observed a slight decrease in the breaking distance that was within the margin of error. The addition of lard or chicken fat had no effect on gel strength, but the addition of soybean oil resulted in better gel strength. Gel strength decreased as the amount of added exogenous lipid increased for all three types of exogenous lipids, which corresponded to the changes in breaking force and breaking distance. There were no significant changes in breaking force, breaking distance, or gel strength among the surimi gels with added composite oils of varying lipid ratios (Figure 2).

The gel strength of surimi gels prepared by water bath and microwave heating was compared. Surimi gels prepared by water bath heating had a breaking force of less than 400 g·cm, but gels prepared by microwave heating had a breaking force of up to 480.92 g·cm. Microwave‐heated surimi gels also had a slightly higher breaking distance than water bath‐heated surimi gels. When compared to water bath heating, the increased breaking force and breaking distance resulted in significantly higher gel strength following microwave heating. This finding is consistent with the experimental results reported by (Cao et al., 2018b). It was also found that the addition of vegetable oil to surimi gels led to an increase in gel brittleness as the amount of added oil increased (Shi et al., 2014). It is, therefore, apparent that microwave heating can be used to boost surimi gel strength by increasing gel brittleness. However, some researchers reported that adding fish oil to a 30% oil content had no effect on the gel properties of surimi (Gani et al., 2018; Okazaki et al., 2002). This is primarily due to the fact that, in addition to the thermal treatment process of surimi gel, which has a significant effect on the gel strength of composite gels, high‐intensity external mechanical emulsification and pretreatment may also alleviate the detrimental effects of lipids on the network structures of surimi gels (Okazaki et al., 2006). Under the impact of high shear force, surimi paste with superior structural homogeneity can be prepared, reducing the effects of lipids on surimi gel properties.

### 3.2 Effects of exogenous lipid addition on the texture profile of surimi gels

The TPA of gels indicates the physical characteristics of gel foods manifested during the chewing process (Pietrowski et al., 2011). It allows the determination of parameters such as hardness, adhesiveness, chewiness, springiness, and resilience, which aids in the understanding of the textural properties of gels. The TPA results on the gels prepared in this study are shown in Table 1. A comparison of TPA results among gels with different added exogenous lipids revealed that gel hardness, chewiness, and gumminess were in the order of lard > chicken fat > soybean oil, although variations in resilience, springiness, cohesiveness, and adhesiveness were not significant. When the amount of added exogenous lipids was increased, the resultant surimi gels showed a significant increase in hardness, chewiness, and gumminess, but a minimal change in resilience, springiness, cohesiveness, and adhesiveness. The impact of composite oils with varying exogenous lipid ratios on the various textural parameters did not differ significantly (Table 2). Although the rate of heating affects the aggregation process of myofibrillar proteins, it may not necessarily cause changes in cohesiveness within gels. Meanwhile, certain chemical reagents that disrupt protein crosslinking may alter the aggregation properties of gels (Pérez‐Mateos et al., 2006).

**TABLE 1:** Effects of different exogenous lipids on the texture profile analysis (TPA) of surimi gels prepared using the two different heating procedures

			0%	3%	6%	9%	12%
Lard	Hardness (g)	WB	1361.08 ± 63.36^Aa^	1408.04 ± 84.01^Ab^	1498.98 ± 74.70^Ac^	1603.91 ± 104.51^Ac^	1729.82 ± 100.52^Ad^
		MW	1394.05 ± 83.71^Aa^	1375.32 ± 36.57^Ab^	1362.82 ± 90.99^Bb^	1385.31 ± 88.39^Ba^	1442.77 ± 160.43^Bc^
	Adhesiveness (g × s)	WB	151.48 ± 49.35^Aa^	172.21 ± 53.66^Ab^	106.78 ± 24.67^Ac^	139.08 ± 53.68^Ad^	130.51 ± 54.06^Ad^
		MW	67.73 ± 20.09^Ba^	67.98 ± 21.38^Ba^	72.18 ± 14.49^Bb^	81.98 ± 19.40^Bc^	47.88 ± 34.61^Bd^
	Springiness (%)	WB	0.89 ± 0.03^Aa^	0.90 ± 0.01^Aa^	0.88 ± 0.03^Aa^	0.89 ± 0.03^Aa^	0.88 ± 0.02^Aa^
		MW	0.90 ± 0.02^Aa^	0.88 ± 0.03^Aa^	0.88 ± 0.03^Aa^	0.86 ± 0.02^Ab^	0.87 ± 0.02^Ab^
	Chewiness	WB	1370.15 ± 96.88^Aa^	1442.51 ± 107.93^Ab^	1498.44 ± 105.94^Ab^	1618.21 ± 133.99^Ac^	1721.15 ± 107.21^Ad^
		MW	1426.44 ± 101.24^Ba^	1375.07 ± 89.11^Bb^	1358.30 ± 136.32^Bb^	1352.96 ± 111.15^Bb^	1423.13 ± 178.61^Ba^
	Gumminess	WB	1537.22 ± 66.65^Aa^	1594.04 ± 97.36^Aa^	1696.00 ± 84.59^Ab^	1809.80 ± 117.88^Ac^	1947.59 ± 107.38^Ad^
		MW	1592.45 ± 88.73^Aa^	1566.05 ± 45.13^Ab^	1549.52 ± 103.50^Bc^	1567.71 ± 95.09^Bb^	1638.54 ± 175.29^Bd^
	Resilience (%)	WB	0.33 ± 0.00^Aa^	0.33 ± 0.01^Aa^	0.32 ± 0.01^Ab^	0.31 ± 0.02^Ac^	0.30 ± 0.02^Ad^
		MW	0.35 ± 0.01^Ba^	0.35 ± 0.01^Ba^	0.33 ± 0.02^Ab^	0.33 ± 0.02^Ab^	0.32 ± 0.01^Ac^
	Cohesiveness	WB	1.13 ± 0.00^Aa^	1.13 ± 0.00^Aa^	1.13 ± 0.00^Aa^	1.13 ± 0.00^Aa^	1.13 ± 0.01^Aa^
		MW	1.14 ± 0.01^Aa^	1.14 ± 0.01^Aa^	1.14 ± 0.01^Aa^	1.13 ± 0.01^Aa^	1.14 ± 0.01^Aa^
Chicken fat	Hardness (g)	WB	1195.19 ± 137.99^Aa^	1287.13 ± 57.80^Ab^	1416.04 ± 27.96^Ac^	1402.87 ± 58.00^Ac^	944.36 ± 29.70^Ad^
		MW	1144.22 ± 71.71^Aa^	1122.99 ± 98.11^Ba^	1210.43 ± 52.15^Bb^	1094.26 ± 37.62^Bc^	1111.75 ± 37.50^Ba^
	Adhesiveness (g × s)	WB	97.71 ± 52.10^Aa^	132.76 ± 35.23^Ab^	106.78 ± 24.67^Aa^	177.17 ± 64.12^Ac^	135.22 ± 12.53^Ab^
		MW	32.64 ± 15.64^Ba^	69.20 ± 35.96^Bb^	85.32 ± 56.84^Bc^	40.53 ± 17.30^Ba^	75.08 ± 39.46^Bb^
	Springiness (%)	WB	0.89 ± 0.03^Aa^	0.88 ± 0.04^Aa^	0.88 ± 0.01^Aa^	0.91 ± 0.02^Ab^	0.89 ± 0.01^Aa^
		MW	0.90 ± 0.02^Aa^	0.90 ± 0.03^Aa^	0.90 ± 0.02^Aa^	0.86 ± 0.01^Bb^	0.89 ± 0.02^Aa^
	Chewiness	WB	1215.72 ± 172.90^Aa^	1292.43 ± 107.44^Aa^	1409.56 ± 48.74^Ab^	1436.12 ± 77.69^Ab^	872.63 ± 167.89^Ac^
		MW	1174.91 ± 93.10^Ba^	1165.30 ± 139.89^Ba^	1251.73 ± 80.43^Bb^	1076.35 ± 35.25^Bc^	1127.84 ± 56.41^Ba^
	Gumminess	WB	1361.05 ± 159.02^Aa^	1457.84 ± 67.66^Ab^	1603.98 ± 33.02^Ac^	1582.49 ± 61.79^Ad^	1070.11 ± 31.68^Ae^
		MW	1315.31 ± 78.22^Aa^	1290.50 ± 112.91^Bb^	1387.56 ± 62.02^Ba^	1254.61 ± 38.87^Bb^	1270.51 ± 42.47^Bb^
	Resilience (%)	WB	0.34 ± 0.00^Aa^	0.33 ± 0.01^Ab^	0.33 ± 0.00^Ab^	0.32 ± 0.00^Ac^	0.35 ± 0.00^Ad^
		MW	0.36 ± 0.01^Ba^	0.37 ± 0.01^Bb^	0.35 ± 0.01^Bc^	0.35 ± 0.01^Bc^	0.35 ± 0.01^Ac^
	Cohesiveness	WB	1.14 ± 0.00^Aa^	1.13 ± 0.00^Ab^	1.13 ± 0.01^Ab^	1.13 ± 0.01^Ab^	1.13 ± 0.00^Ab^
		MW	1.15 ± 0.00^Ba^	1.15 ± 0.01^Ba^	1.15 ± 0.01^Ba^	1.15 ± 0.01^Ba^	1.14 ± 0.01^Ab^
Soybean oil	Hardness (g)	WB	994.32 ± 68.27^Aa^	1020.56 ± 7.49^Ab^	965.59 ± 15.47^Ac^	973.09 ± 29.24^Ac^	1399.05 ± 40.90^Ad^
		MW	764.48 ± 55.49^Ba^	773.23 ± 63.25^Ba^	834.43 ± 19.13^Ab^	796.96 ± 84.65^Ba^	715.77 ± 86.58^Ac^
	Adhesiveness (g × s)	WB	101.51 ± 28.27^Aa^	149.57 ± 17.93^Ab^	142.26 ± 8.14^Ab^	115.64 ± 31.10^Ac^	128.40 ± 16.37^Ad^
		MW	56.53 ± 33.81^Ba^	62.85 ± 37.58^Bb^	86.00 ± 37.28^Bc^	61.11 ± 50.95^Bb^	39.43 ± 19.93^Bd^
	Springiness (%)	WB	0.89 ± 0.03^Aa^	0.90 ± 0.01^Aa^	0.90 ± 0.01^Aa^	0.90 ± 0.01^Aa^	0.88 ± 0.02^Aa^
		MW	0.87 ± 0.03^Aa^	0.88 ± 0.04^Aa^	0.91 ± 0.01^Ab^	0.87 ± 0.02^Ba^	0.86 ± 0.04^Aa^
	Chewiness	WB	1005.45 ± 97.89^Aa^	1037.77 ± 12.85^Ab^	977.92 ± 21.05^Ac^	989.64 ± 38.83^Ac^	1381.85 ± 54.24^Ad^
		MW	758.19 ± 75.42^Ba^	782.45 ± 88.07^Bb^	866.17 ± 27.93^Bc^	798.86 ± 96.74^Bb^	707.93 ± 110.85^Bd^
	Gumminess	WB	1130.41 ± 73.12^Aa^	1157.69 ± 6.45^Aa^	1091.51 ± 17.25^Ab^	1103.55 ± 30.22^Ab^	1574.99 ± 44.65^Ac^
		MW	872.69 ± 61.12^Ba^	883.19 ± 64.74^Ba^	953.26 ± 26.15^Bb^	914.86 ± 90.19^Bc^	822.93 ± 94.15^Bd^
	Resilience (%)	WB	0.36 ± 0.02^Aa^	0.35 ± 0.01^Ab^	0.35 ± 0.00^Ab^	0.35 ± 0.01^Ab^	0.31 ± 0.01^Ac^
		MW	0.37 ± 0.01^Aa^	0.36 ± 0.01^Ab^	0.37 ± 0.01^Ba^	0.36 ± 0.01^Ab^	0.37 ± 0.01^Ba^
	Cohesiveness	WB	1.14 ± 0.01^Aa^	1.13 ± 0.01^Ab^	1.13 ± 0.00^Ab^	1.13 ± 0.01^Ab^	1.13 ± 0.01^Ab^
		MW	1.14 ± 0.01^Aa^	1.14 ± 0.01^Aa^	1.14 ± 0.00^Ba^	1.15 ± 0.01^Bb^	1.15 ± 0.01^Bb^

*Note*: The data are expressed as mean ± standard deviations (*n* = 5); 0%: control; 3%, 6%, 9%, and 12%: supplementation with 3%, 6%, 9%, and 12% exogenous lipids; uppercase letters indicate significant difference (*p* < .05) between different heating treatments; lowercase letters indicate the difference between gels with different exogenous lipids contents.

**TABLE 2:** Effects of different composite oils on the texture profile analysis (TPA) of surimi gels prepared using the two different heating procedures

		Hardness (g)	Adhesiveness (g × s)	Springiness (%)	Gumminess	Chewiness	Resilience (%)	Cohesiveness
5∶2∶2	WB	1336.10 ± 65.02^Aa^	152.04 ± 59.33^Aa^	0.91 ± 0.01^Aa^	1369.26 ± 67.32^Aa^	1511.42 ± 66.92^Aa^	0.33 ± 0.01^Aa^	1.13 ± 0.00^Aa^
	MW	1129.23 ± 39.34^Ba^	93.14 ± 27.86^Ba^	0.89 ± 0.03^Aa^	1144.19 ± 78.14^Ba^	1283.15 ± 48.83^Ba^	0.34 ± 0.02^Aa^	1.14 ± 0.01^Aa^
2∶5∶2	WB	1367.07 ± 79.13^Aa^	138.46 ± 39.47^Ab^	0.89 ± 0.02^Aa^	1374.61 ± 106.59^Aa^	1551.69 ± 84.54^Aa^	0.34 ± 0.01^Ab^	1.13 ± 0.00^Aa^
	MW	1204.18 ± 64.67^Ab^	78.09 ± 16.84^Bb^	0.88 ± 0.02^Aa^	1204.03 ± 77.56^Bb^	1371.57 ± 68.85^Bb^	0.35 ± 0.02^Ab^	1.14 ± 0.01^Aa^
2∶2∶5	WB	1420.03 ± 70.43^Ab^	163.48 ± 53.30^Aa^	0.92 ± 0.01^Aa^	1475.75 ± 78.32^Aa^	1611.92 ± 74.97^Ab^	0.34 ± 0.01^Ab^	1.13 ± 0.01^Aa^
	MW	1233.16 ± 104.65^Bb^	67.75 ± 37.84^Bc^	0.90 ± 0.02^Aa^	1278.80 ± 131.55^Bc^	1414.91 ± 116.81^Bc^	0.36 ± 0.02^Bc^	1.15 ± 0.01^Bb^
3∶3∶3	WB	1439.02 ± 16.82^Ab^	120.99 ± 48.80^Ab^	0.90 ± 0.01^Aa^	1470.19 ± 34.90^Aa^	1637.43 ± 21.59^Ac^	0.34 ± 0.02^Ab^	1.14 ± 0.01^Ab^
	MW	1237.91 ± 50.03^Bb^	95.96 ± 19.63^Ba^	0.90 ± 0.01^Aa^	1275.39 ± 51.45^Bc^	1412.90 ± 52.29^Bc^	0.36 ± 0.02^Bc^	1.14 ± 0.00^Aa^

*Note*: The data are expressed as mean ± standard deviations (*n* = 5); uppercase letters indicate significant difference (*p* < .05) between different heating treatments; lowercase letters indicate the difference between gels with different lipid ratios.

When the textural properties of surimi gels prepared by water bath heating and microwave heating were compared, we found that hardness, chewiness, gumminess, and adhesiveness were significantly lower in microwave‐heated surimi gels than in water bath‐heated gels, whereas resilience, springiness, and cohesiveness did not differ significantly. For instance, the hardness of surimi gel containing 6% lard was 1362.82 g after microwave heating and 1498.98 g after water bath heating. These results indicated that microwave heating treatment is capable of improving the gel properties of surimi gel. This is in line with the findings of (Debusca et al., 2013), who reported that microwave heating produced gels with a softer texture.

### 3.3 Effects of exogenous lipid addition on the whiteness and water holding capacity of surimi gels

Protein structure and denaturation are two factors that influence the whiteness of surimi gels (Sun & Holley, 2011). Regardless of the heating method or kind of exogenous lipid used, our experimental results show that surimi gel whiteness increased significantly with increasing amounts of added exogenous lipid (Table 3). Such an increase in gel whiteness may be attributed to changes in the degree of light scattering caused by emulsification in oil–water mixtures. After conventional water bath heating, the order of gel whiteness was chicken fat > soybean oil > lard, and after microwave heating, the order was soybean oil > chicken fat > lard, which provides a reference for the enhancement of sensory qualities of surimi products during surimi processing. Whiteness did not differ significantly among those heated using different methods or those with added composite oils of different exogenous lipid ratios (Table 4).

**TABLE 3:** Effects of different exogenous lipids on the whiteness of surimi gels prepared using the two different heating procedures

			0%	3%	6%	9%	12%
Lard	L*	WB	83.25 ± 0.07^Aa^	83.61 ± 0.15^Aa^	84.24 ± 0.08^Ab^	85.16 ± 0.01^Ac^	85.93 ± 0.05^Ac^
		MW	80.77 ± 0.01^Ba^	81.96 ± 0.04^Bb^	83.42 ± 0.07^Ac^	84.56 ± 0.11^Ad^	86.97 ± 0.03^Ae^
	a*	WB	−1.33 ± 0.00^Aa^	−1.31 ± 0.05^Aa^	−1.23 ± 0.02^Ab^	−1.11 ± 0.03^Ac^	−1.07 ± 0.02^Ac^
		MW	−1.66 ± 0.03^Ba^	−1.57 ± 0.03^Bb^	−1.53 ± 0.02^Bb^	−1.36 ± 0.02^Bc^	−1.20 ± 0.02^Bd^
	b*	WB	7.73 ± 0.05^Aa^	7.80 ± 0.21^Aa^	7.80 ± 0.05^Aa^	8.15 ± 0.04^Ab^	7.86 ± 0.01^Aa^
		MW	7.07 ± 0.01^Aa^	7.56 ± 0.02^Ab^	7.80 ± 0.02^Ac^	7.56 ± 0.05^Bb^	7.30 ± 0.03^Ad^
	Whiteness	WB	81.51 ± 0.04^Aa^	81.80 ± 0.06^Aa^	82.37 ± 0.07^Ab^	83.03 ± 0.03^Ac^	83.85 ± 0.04^Ac^
		MW	79.45 ± 0.09^Ba^	80.38 ± 0.03^Bb^	81.62 ± 0.07^Bc^	82.76 ± 0.12^Bd^	85.02 ± 0.04^Be^
Chicken fat	L*	WB	84.06 ± 0.08^Aa^	86.26 ± 0.03^Ab^	87.82 ± 0.04^Ac^	88.86 ± 0.06^Ad^	89.49 ± 0.05^Ae^
		MW	80.84 ± 0.01^Ba^	83.97 ± 0.06^Bb^	85.54 ± 0.12^Bc^	86.97 ± 0.06^Bd^	87.94 ± 0.01^Be^
	a*	WB	−1.02 ± 0.02^Aa^	−0.84 ± 0.02^Ab^	−0.69 ± 0.03^Ac^	−0.67 ± 0.02^Ac^	−0.62 ± 0.01^Ac^
		MW	−1.63 ± 0.02^Ba^	−1.32 ± 0.01^Bb^	−1.21 ± 0.01^Bc^	−1.12 ± 0.02^Bd^	−1.05 ± 0.04^Be^
	b*	WB	8.34 ± 0.02^Aa^	7.88 ± 0.03^Ab^	7.98 ± 0.05^Ac^	7.26 ± 0.03^Ad^	7.45 ± 0.03^Ae^
		MW	7.34 ± 0.01^Ba^	7.57 ± 0.01^Ab^	7.28 ± 0.12^Ac^	7.17 ± 0.02^Ad^	6.92 ± 0.00^Be^
	Whiteness	WB	81.98 ± 0.08^Aa^	84.14 ± 0.04^Ab^	85.42 ± 0.04^Ac^	86.68 ± 0.06^Ad^	87.11 ± 0.05^Ae^
		MW	79.42 ± 0.06^Ba^	82.23 ± 0.05^Bb^	83.76 ± 0.05^Bc^	85.08 ± 0.04^Bd^	86.06 ± 0.01^Be^
Soybean oil	L*	WB	83.84 ± 0.06^Aa^	85.75 ± 0.03^Ab^	86.82 ± 0.06^Ac^	87.97 ± 0.05^Ad^	86.97 ± 0.04^Ac^
		MW	81.92 ± 0.01^Ba^	84.57 ± 0.12^Bb^	86.04 ± 0.19^Ac^	87.34 ± 0.14^Ad^	87.97 ± 0.19^Bd^
	a*	WB	−1.44 ± 0.02^Aa^	−1.26 ± 0.00^Ab^	−1.21 ± 0.01^Ab^	−1.09 ± 0.00^Ac^	−1.20 ± 0.01^Ab^
		MW	−1.71 ± 0.02^Ba^	−1.47 ± 0.01^Bb^	−1.31 ± 0.03^Bc^	−1.26 ± 0.01^Bc^	−1.27 ± 0.03^Ac^
	b*	WB	8.06 ± 0.09^Aa^	8.10 ± 0.02^Aa^	7.81 ± 0.04^Ab^	7.69 ± 0.04^Ac^	7.30 ± 0.02^Ad^
		MW	7.56 ± 0.06^Ba^	8.01 ± 0.03^Ab^	7.69 ± 0.03^Bc^	7.83 ± 0.01^Bd^	7.57 ± 0.03^Be^
	Whiteness	WB	81.89 ± 0.03^Aa^	83.56 ± 0.02^Ab^	84.63 ± 0.07^Ac^	85.68 ± 0.06^Ad^	85.02 ± 0.04^Ad^
		MW	80.33 ± 0.02^Ba^	82.55 ± 0.12^Bb^	84.01 ± 0.17^Ac^	85.07 ± 0.13^Ad^	85.73 ± 0.19^Ad^

*Note*: The data are expressed as mean ± standard deviations (*n* = 5); 0%: control; 3%, 6%, 9%, and 12%: supplementation with 3%, 6%, 9%, and 12% exogenous lipids; uppercase letters indicate significant difference (*p* < .05) between different heating treatments; lowercase letters indicate the difference between gels with different exogenous lipid contents.

**TABLE 4:** Effects of composite oils on the whiteness of surimi gels prepared using the two different heating procedures

		L*	a*	b*	Whiteness
5∶2∶2	WB	86.00 ± 0.22^Aa^	−1.04 ± 0.03^Aa^	7.73 ± 0.08^Aa^	83.98 ± 0.17^Aa^
	MW	84.90 ± 0.19^Ba^	−1.31 ± 0.05^Ba^	7.76 ± 0.16^Aa^	82.97 ± 0.15^Ba^
2∶5∶2	WB	86.84 ± 0.28^Ab^	−0.92 ± 0.03^Ab^	7.78 ± 0.07^Aa^	84.68 ± 0.25^Ab^
	MW	85.98 ± 0.21^Bb^	−1.21 ± 0.02^Bb^	7.76 ± 0.07^Aa^	83.93 ± 0.18^Bb^
2∶2∶5	WB	87.29 ± 0.06^Ac^	−0.85 ± 0.02^Ac^	7.38 ± 0.04^Ab^	85.28 ± 0.03^Ac^
	MW	86.55 ± 0.13^Bc^	−1.15 ± 0.06^Bb^	7.35 ± 0.04^Bb^	84.63 ± 0.10^Bc^
3∶3∶3	WB	87.24 ± 0.02^Ac^	−0.84 ± 0.01^Ac^	7.35 ± 0.08^Ab^	85.25 ± 0.04^Ac^
	MW	86.41 ± 0.04^Bc^	−1.18 ± 0.01^Bb^	7.29 ± 0.02^Bc^	84.54 ± 0.04^Bc^

*Note*: The data are expressed as mean ± standard deviations (*n* = 5); uppercase letters indicate significant difference (*p* < .05) between different heating treatments; lowercase letters indicate the difference between gels with different lipid ratios.

**FIGURE 3:**
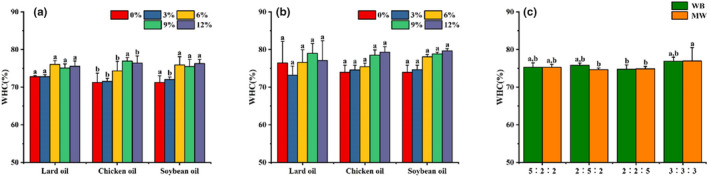
The effect of different exogenous lipids on the water holding capacity (WHC) of surimi gels prepared using water bath heating (a) and microwave heating (b). The effect of composite oils (c) on the WHC of surimi gels prepared using the two different heating procedures. (a)(b): Different letters within each color bar denote significant difference (*p* < .05) for 0%, 3%, 6%, 9%, and 12% addition of different exogenous lipids, respectively. (c): Different letters within each color bar denote significant difference (*p* < .05) between different heating treatments

Water holding capacity (WHC) reflects the degree of tightness of protein–water bonding within the surimi gel, with higher WHC values generally indicating greater chewiness (Cao et al., 2018a). In our experiment, WHC did not differ significantly between surimi gels prepared by different heating methods or among surimi gels with different added exogenous lipids (Figure 3). An increase in the amount of added exogenous lipid caused WHC to increase slightly, although the increase was not statistically significant. Similarly, (Fukushima et al., 2007) also reported an increase in the WHC of surimi gels as the amount of added fish oil increased. This indicates the formation of a homogenous, stable system after emulsification of the fish oil–surimi mixture, with the presence of oil droplets preventing water loss. This finding is in agreement with the findings from our investigation of gel strength in Section 3.1, which revealed that microwave heating significantly increased surimi gel strength. The three‐dimensional (3D) protein structures within the surimi gel can retain a greater amount of water as the gel strength increases, resulting in a reduction in the amount of water that can be extracted. (Fu et al., 2019) observed that microwave heating significantly increased the WHC of silver carp surimi gels. The presence of hydrocolloid substances also significantly enhances the WHC of surimi gels prepared by microwave heating (Fu et al., 2012). Composite oils with different ratios of exogenous lipids did not lead to significant differences in the WHC of the resultant surimi gels (Ji et al., 2017), with the WHC values remaining at approximately 75%. This indicates that the addition of different types of composite oils did not exert a considerable influence on surimi gel properties. Therefore, the addition of multiple types of lipids during actual surimi production is unlikely to result in significant changes in surimi gel quality.

### 3.4 Effects of exogenous lipid addition on the microscopic structures of surimi gels

**FIGURE 4:**
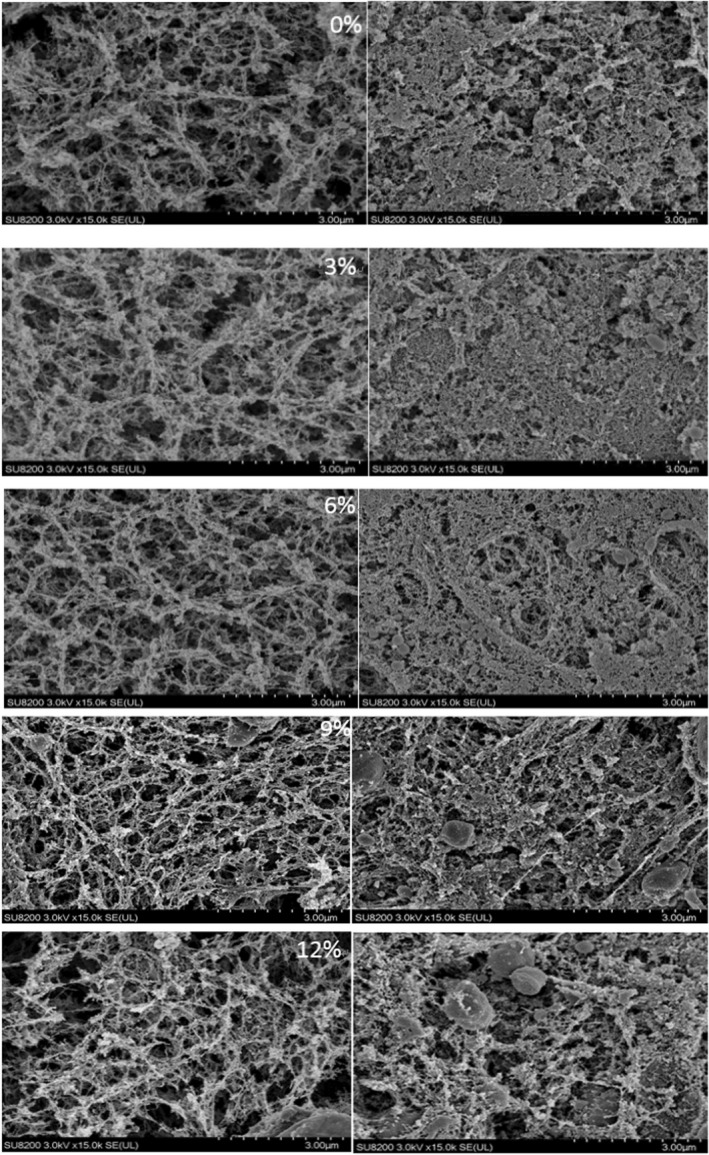
The effect of lard on the microstructure of surimi gels prepared using water bath heating (left) and microwave heating (right). Micrographs were taken at a magnification of 15,000 × to analyze the morphological characteristics

To determine the effects of fat globules on gel networks, we analyzed the microscopic structures of surimi gels with added lard by SEM. Figure 4 shows the SEM images of surimi gels containing different mass fractions of lard prepared by two different heating methods. Surimi gels with 3% lard exhibited gel structures with higher density and smaller pore size compared with those without added exogenous lipids. However, as the amount of added lard increased, we observed that the degree of protein crosslinking and density of the gel structure reduced, while pore size increased. This indicates that the oil droplets prevented protein gels from aggregating, which is consistent with the results reported by (Gani & Benjakul, 2019) and is also in agreement with the findings explained in Section 3.1. Therefore, it is evident that the addition of exogenous lipids interferes with protein gel formation. The degree of network homogeneity in surimi gels is affected by the oil droplet size. It was reported that fish oil droplet size decreased with an increase in rotation speed during chopping (Okazaki et al., 2006). A comparison of the SEM images of surimi gels obtained by microwave heating and water bath heating revealed that the protein network structures of surimi gels prepared by water bath heating had lower density and larger pores compared with surimi gels prepared by microwave heating, which is in agreement with the findings from the investigation of gel strength. This may be explained by the enlargement of exposed hydrophobic regions of myosin molecules caused by the rapid nature of microwave heating. As a result, the interactions between fish oil and proteins were altered, obstructing the formation of interfacial protein films on the surfaces of oil droplets, and ultimately, affecting the degree of aggregation in gel networks. Similarly, (Cao et al., 2020) also observed that microwave heating increased the opacity and surface hydrophobicity of myosin and promoted protein molecule aggregation by altering the secondary structures and unfolding of myosin, thereby increasing the gel strength of myofibrillar proteins.

### 3.5 Fatty acid analysis of exogenous lipids

The fatty acid components of the exogenous lipids employed in this investigation were analyzed by GC–MS. As shown in Table 5, the fatty acids of the various types of lipids were concentrated within the C14–C20 range. The most prevalent fatty acid in lard was oleic acid (accounting for 34.93% of the fatty acid content), followed by palmitic acid and stearic acid. Chicken fat was mostly composed of oleic and palmitic acids (which, together, accounted for 69.88% of the fatty acid content), but soybean oil's fatty acids were dominated by linoleic acid and included much more polyunsaturated fatty acids than lard and chicken fat. The results of our quality analysis of surimi gels with different added exogenous lipids revealed that the gel quality of surimi gels with added soybean oil was slightly higher than those of gels with added lard and chicken fat. Among the surimi gels with added composite oil, those with a higher ratio of soybean oil had slightly higher gel quality. Therefore, it can be deduced that the enhancement of surimi gel quality by soybean oil may be associated with its high polyunsaturated fatty acid content.

**TABLE 5:** The main fatty acid composition of exogenous lipids

Fatty acid composition	The relative fatty acid content of fatty acid^a^/%						
	Lard	Chicken oil	Soybean oil	5:2:2	2:5:2	2:2:5	3:3:3
C14:0	2.10	1.44	0.00	1.40	1.13	0.87	1.22
C16:0	21.39	21.74	12.04	14.45	20.76	16.27	18.65
C16:1	3.08	6.91	0.00	3.53	5.15	2.81	5.12
C17:0	0.98	0.00	0.00	0.80	0.00	0.00	0.55
C18:0	18.97	0.40	8.50	12.10	9.33	10.86	9.28
C18:1	34.93	48.14	24.45	44.75	36.47	30.98	36.07
C18:2	13.32	18.41	37.65	16.39	21.04	28.95	23.46
C18:3	0.99	1.31	10.18	3.00	3.29	6.47	4.11
C20:0	0.66	0.00	1.23	0.70	0.50	0.89	0.68
C20:1	2.15	1.65	2.75	1.84	1.59	1.35	2.28
C20:2	1.43	0.00	3.20	1.04	0.71	0.55	0.56
∑SFA	44.10	23.58	21.77	29.45	31.72	28.89	30.38
∑MUFA	40.16	56.70	27.20	50.12	43.21	35.14	42.49
∑PUFA	15.74	19.72	51.03	20.43	25.07	35.97	27.13

a The relative content is the percentage of peak area of gas chromatography (GC) of each component.

## 4 CONCLUSION

In this study, we investigated the effects of surimi gel properties with exogenous lipids added, including lard, chicken fat, and soybean oil. According to our results, the addition of an appropriate amount of exogenous lipid improved the gel strength. However, increasing the amount of exogenous lipid resulted in a drop of gel strength and a slight increase in the WHC. In addition, whiteness increased significantly with an increase in the amount of added exogenous lipid, which enhanced the sensory quality of the surimi gels. Surimi gel properties were not significantly changed when composite oils with varying ratios of exogenous lipids were used. An analysis of the fatty acid compositions of the exogenous lipids suggested that the enhancement of surimi gel quality by soybean oil may be associated with its high polyunsaturated fatty acid content. Compared with surimi gels prepared by conventional water bath heating, surimi gels prepared by microwave heating exhibited a significant improvement in gel characteristics. This illustrates that microwave heating may be used to achieve effective thermal gelation during the processing of surimi products containing exogenous lipids.

## DATA AVAILABILITY STATMENT

The data that support the finding of this study are available from the corresponding author upon reasonale request.
